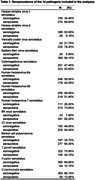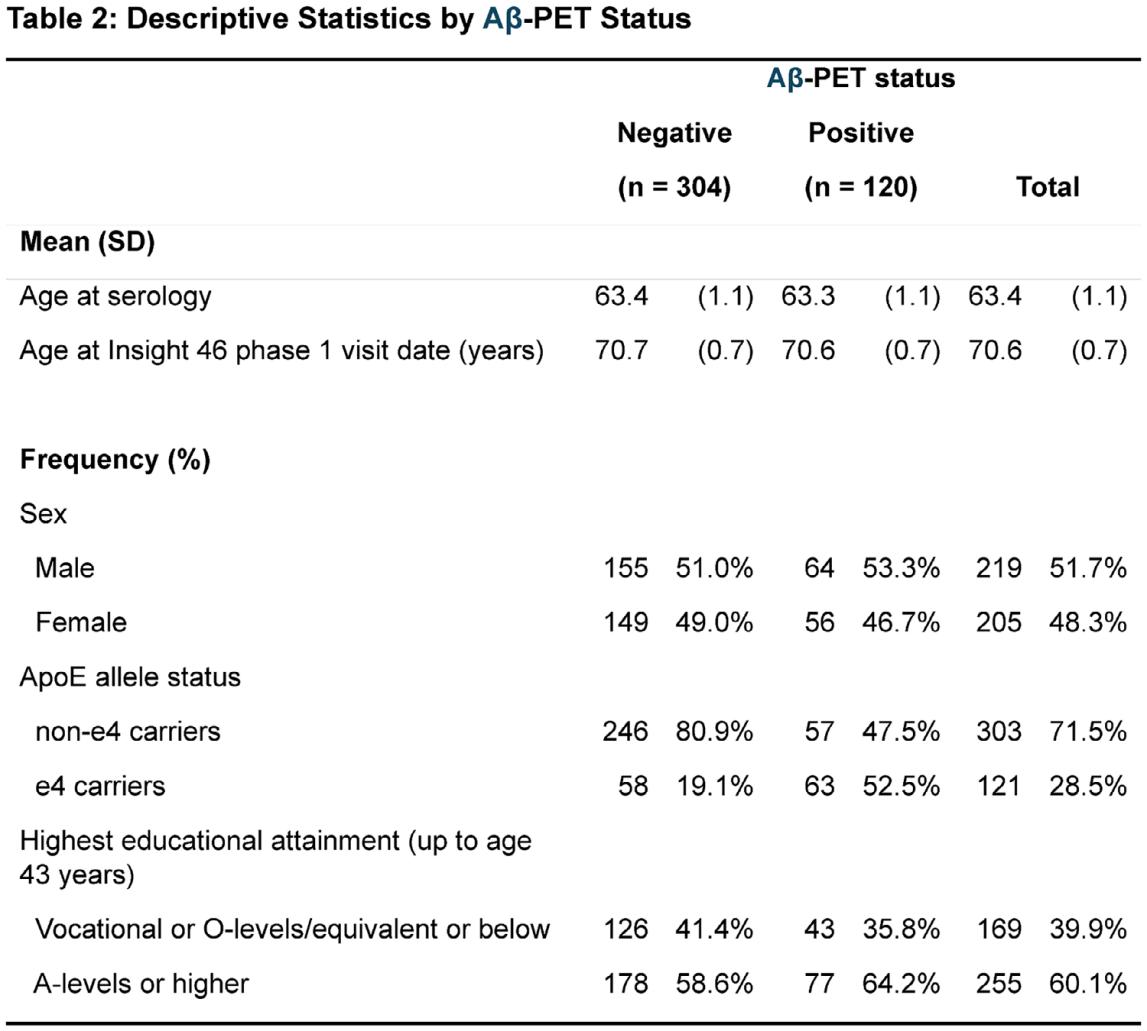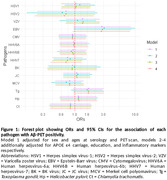# Associations of several common infections with amyloid‐β pathology *in vivo*: a population‐based study

**DOI:** 10.1002/alz.092178

**Published:** 2025-01-09

**Authors:** Caterina Felici, Rebecca E Green, Charlotte Warren‐Gash, Julia Butt, Tim Waterboer, Insight 46 study Team, Alun D Hughes, Jonathan M Schott, Ashvini Keshavan, Nishi Chaturvedi, Marcus Richards, Dylan M Williams

**Affiliations:** ^1^ MRC Unit for Lifelong Health & Ageing at UCL, London United Kingdom; ^2^ London School of Hygiene and Tropical Medicine, London United Kingdom; ^3^ Division of Infections and Cancer Epidemiology, German Cancer Research Center (DKFZ), Heidelberg Germany; ^4^ Dementia Research Centre, UCL Queen Square Institute of Neurology, London United Kingdom

## Abstract

**Background:**

Associations of common infections with Alzheimer’s disease have been reported, but potential mechanisms underlying these relationships are unclear. A hypothesised mechanism is amyloid‐beta (Aβ) aggregation as a defense mechanism in response to infection, with subsequent tau accumulation. However, no studies have assessed associations of infections with cerebral Aβ and tau pathology *in vivo*. We investigated relationships between serum antibodies to common infections and Aβ pathology quantified using PET in the Insight 46 neuroimaging cohort.

**Method:**

Circulating antibody levels against 14 pathogens, measured at age 60‐64, were modelled as pathogen serostatus, cumulative pathogen burden, and seroreactivity tertiles. Their associations with Aβ‐PET positivity, assessed 7.3(±1.3) years after serology measurement, were modelled using multivariable logistic regression. Model 1 adjusted for sex and ages at serology and PET scan, models 2‐4 additionally adjusted for *APOE* ε4 carriage, education, and inflammatory markers respectively. We tested for interactions in associations by *APOE* ε4 carriage and education, and interactions between herpes simplex virus 1 and both cytomegalovirus and varicella‐zoster virus. Analyses were repeated using Aβ‐PET deposition as a continuous variable.

**Result:**

424 individuals had serology and Aβ‐PET data (Tables 1 and 2). Individuals seropositive to Epstein‐Barr virus (EBV) had higher odds of Aβ‐PET positivity in all models, with a large point estimate but wide confidence intervals including null (Model 2: OR 3.11; 95% CI: 0.96,10.09; Figure 1). Similar patterns were observed when analysing Aβ‐PET continuously (Model 2: 22% higher burden; 95% CI: ‐4, 47%). Individuals seropositive to *Toxoplasma gondii* (T.gondii) had a higher likelihood of Aβ‐PET positivity with large confidence intervals including null (Model 2: OR 1.60; 95% CI: 0.87, 2.94; Figure 1). There was modest evidence for an interaction between human herpesvirus 7 (HHV7) and education in relation to Aβ‐PET positivity (lower Aβ risk among HHV7 seropositives with higher education). No other pronounced findings were observed.

**Conclusion:**

Results suggest potentially strong associations of EBV and T.gondii seropositivity with Aβ burden, however, the large CIs including null suggest the study lacked power to generate precise estimates. Findings warrant replication in other larger cohorts and with other neurobiomarkers. We plan to expand our analyses to plasma p‐tau217 as another outcome (N≤1454).